# The effect of enhanced glycolysis on cardiac aging

**DOI:** 10.1007/s11357-025-01656-z

**Published:** 2025-05-01

**Authors:** Anna Faakye, Kylene M. Harold, Satoshi Matsuzaki, Atul Pranay, Maria F. Mendez Garcia, Brooke L. Loveland, Sandra N. Rigsby, Frederick F. Peelor, Craig Eyster, Benjamin F. Miller, Timothy M. Griffin, Michael Kinter, Ying Ann Chiao, Kenneth M. Humphries

**Affiliations:** 1 Aging and Metabolism Research Program, Oklahoma Medical Research Foundation, 825 N.E. 13 th Street, Oklahoma City, OK USA; 2 Department of Biochemistry and Molecular Physiology, University of Oklahoma Health Sciences Center, Oklahoma City, OK USA; 3 Oklahoma City Veterans Association Medical Center, Oklahoma City, OK USA

**Keywords:** Cardiac aging, Glycolysis, Mitochondria, Oxidative stress, Proteostasis

## Abstract

Cardiac aging is associated with metabolic changes, including an increased reliance on glycolysis, and an increased susceptibility to cardiovascular diseases. This study explores the relationship between enhanced cardiac glycolysis and aging using the Glyco^Hi^ mouse model, characterized by constitutively elevated glycolysis. We compared cardiac function, metabolism, mitochondrial performance, and hallmarks of aging between aged (21 and 24 months) Glyco^Hi^ and wild-type (WT) mice across sexes. Our findings reveal modest reductions in cardiac function in aged Glyco^Hi^ mice compared to WT mice, with sex-specific differences in heart size and collagen concentration. Female Glyco^Hi^ hearts exhibited hypertrophy without fibrosis, while males showed elevated collagen levels. Whole-body metabolic assessments revealed similar energy expenditure and respiratory patterns across genotypes, with females displaying less circadian-associated variation in metabolism. Mitochondrial analyses showed that aged Glyco^Hi^ hearts maintained metabolic adaptations favoring glycolysis but did not exhibit significant bioenergetic dysfunction or oxidative stress. Pyruvate dehydrogenase activity, initially elevated in younger Glyco^Hi^ hearts, normalized to WT levels with age. Proteomic and metabolomic analyses highlighted distinct profiles between genotypes, with Glyco^Hi^ hearts exhibiting increased glycolytic enzyme levels and reduced abundance of fatty acid oxidation proteins. Despite these differences, indicators of oxidative stress, proteostasis, and cellular senescence were comparable between genotypes, suggesting no acceleration of aging-related dysfunction. This study demonstrates that increased cardiac glycolysis alone does not suffice to drive accelerated cardiac aging. Instead, metabolic and functional changes in aged Glyco^Hi^ hearts reflect adaptations rather than pathological declines, providing insights into potential metabolic targets for interventions against cardiac aging.

**Supplementary Information:**

The online version contains supplementary material available at 10.1007/s11357-025-01656-z.

## Introduction

Aging causes a progressive decrease in cardiac function and increases the risk for cardiovascular diseases. The underlying causes are complex, but identifying the molecular changes that underly the aged cardiac phenotype opens the door for potential therapeutic interventions [1, 2]. The aged heart is metabolically distinct from the young heart in several aspects. The young heart relies primarily on fatty acid oxidation as the primary energy source. It also has robust metabolic flexibility to switch to glucose oxidation and other available substrates. The aged heart still relies primarily on fatty acid oxidation but also displays enhanced glucose oxidation and a decreased metabolic flexibility [3]. There are also differences in mitochondrial function. The aged heart mitochondria show decreased respiratory function and increased ROS production [3]. Other hallmarks of aging are also apparent in the aged heart, including changes in proteostasis, which can affect mitochondrial function [4, 5]. Collectively, these molecular changes predispose the heart to a loss of function and increased susceptibility to stress-induced damage.

Phosphofructokinase- 2/fructose bisphosphatase- 2 (PFKFB) is a bifunctional enzyme family that controls the production and degradation of fructose- 2,6-bisphosphate (F- 2,6-BP). The kinase activity of PKFB (also known as PFK- 2) is responsible for generating F- 2,6-BP, which is a potent allosteric activator of phosphofructokinase- 1, a rate-limiting enzyme of glycolysis [6]. The Glyco^Hi^ mouse is a cardiac-specific transgenic model of increased cardiac glycolysis [7–9]. Glyco^Hi^ mice express a mutated form of the PFKFB1 isoform of the PFK- 2 family that lacks fructose bisphosphatase- 2 activity. The transgene constitutively generates F- 2,6-BP which results in elevated rates of glycolysis at the expense of decreased fatty acid oxidation. We have previously shown that adult Glyco^Hi^ mice have enhanced PDH activity, indicative of increased glucose oxidation [9, 10]. Increasing glycolysis through the activation of this key regulatory point (the PFK- 2/PFK- 1 regulatory nexus) is sufficient to drive the uptake of glucose [11, 12].

Treatments that target cardiac aging may do so by affecting cardiac metabolism. For example, the beneficial effects of rapamycin on the aged heart are mediated in part by a reversal of the metabolic phenotype from glycolysis to FAO [13]. However, reciprocally, it is unclear whether increased cardiac glucose metabolism is sufficient to drive cardiac aging. The goal of this study was to address this question and determine whether increased cardiac glycolysis via the constitutive activation of the PFK- 2/PFK- 1 regulatory nexus is sufficient to accelerate cardiac aging. We hypothesized that Glyco^Hi^ mice would be spared from an accelerated aging phenotype, as we have previously shown Glyco^Hi^ mice at a younger age (9–11 months) have largely preserved function and an increased resilience to metabolic stress [9]. We compare wild type and Glyco^Hi^ mice cardiac function, whole-body metabolism, cardiac metabolism, and hallmarks of aging. We show that despite their enhanced cardiac glycolysis, there is not an accelerated cardiac aging phenotype.

## Methods

### Animals

All animal procedures were approved by the Oklahoma Medical Research Foundation Animal Care and Use Committee. Animals were maintained on a standard ad libitum lab chow diet. Littermates of mature adult transgenic Glyco^Hi^ and WT mice on the FVB/NJ background (21–24 months old) were used in this study. Mice were group-housed and maintained on a 14-h light/dark cycle (light from 06:00 to 20:00). Glyco^Hi^ mice were obtained from the Epstein lab at the University of Louisville, and the development of these transgenic mice has previously been described [7]. Briefly, Glyco^Hi^ mice have cardiac-specific expression of a mutated, phosphatase-deficient, PFKFB1 bound to the α-myosin heavy-chain promoter. The result is a constitutive increase in fructose- 2,6-bisphosphate and enhanced glycolysis. Mice were euthanized by cervical dislocation and experiments were started at the same time each day.

### Echocardiography

Echocardiographic measurement was performed as previously described [14]. Briefly, a cohort of male and female mice were anesthetized using 2–2.5% isoflurane for induction and 1.0% isoflurane for maintenance. The Siemens Acuson CV- 70 system with a 13-MHz probe was utilized for image acquisition and analysis. Motion mode (M-mode) in parasternal long axis view and pulsed wave Doppler imaging in apical 4-chamber view were used to measure systolic and diastolic function, respectively. Myocardial performance index was calculated as (isovolumic contraction time + isovolumic relaxation time)/ejection time.

### Indirect calorimetry

Energy expenditure was measured by indirect calorimetry as described previously [15, 16]. Animals were acclimated to the testing cages and metabolic cabinet for 48 h before data collection with ad libitum access to food and water. After acclimation, oxygen consumption and carbon dioxide production were measured using a multiple animal respirometry system (MARS) (Sable Systems, Las Vegas, NV, USA). 10‐min/animal averages were collected hourly over a continuous 20‐h period. The respiratory exchange ratio was calculated as the ratio of the average carbon dioxide produced to oxygen consumed over this time period. Energy expenditure was calculated as described using the caloric equivalent of oxygen [17]. Data were subdivided into the light or dark phase time periods to evaluate potential differences in metabolic responses associated with different activity levels. Rates of energy expenditure were normalized to either total body mass or lean body mass. Body mass was measured at the time of introduction to the metabolic cages. Body composition (i.e., lean and fat mass) was measured by quantitative magnetic resonance (EchoMRI).

### Mitochondrial respiration

Mitochondria were isolated by differential centrifugation and respirometry was performed as previously described [18]. Following isolation and suspension, mitochondria were further diluted to 0.25 mg/mL in OXPHOS buffer (210 mM mannitol, 70 mM sucrose, 10 mM MOPS, and 5 mM K_2_HPO_4_, pH 7.4), with 0.5 mg/mL BSA. Respiration (State 2) was initiated with either 0.1-mM pyruvate and 1.0-mM malate, or 30-µM palmitoyl carnitine and 1.0-mM malate. A fluorescence lifetime-based dissolved oxygen monitoring system (Instech) was utilized to quantify oxygen consumption. After 2 min, 0.5-mM ADP was added to induce state 3 respiration. State 4 respiration was calculated as the slower, linear rate of oxygen consumption that occurred approximately 5 min after the initiation of state 3.

### Pyruvate dehydrogenase activity assay

Pyruvate dehydrogenase activity was assayed as described previously [14]. Briefly, freshly isolated mitochondria were suspended in mitochondrial isolation buffer. The mitochondrial suspension was diluted 3:100 in OXPHOS buffer (see above) and then further diluted 1:5 in 25 mM MOPS with 0.05% Triton X- 100. Activity was measured on an Agilent 8453 UV/Vis spectrophotometer on kinetic mode (340 nm absorbance every 5 s for 275 s) upon the addition of 1.0-mM NAD^+^, 2.5-mM pyruvate, 200-µM thiamine pyrophosphate, 100-µM CoASH, and 5.0-mM MgCl_2_. Activities were normalized to protein concentrations (BCA).

### Metabolomic analysis using LC–MS and GC–MS

LC–MS targeted and GC–MS semi-targeted metabolomic analyses were performed from a single preparation as previously described [14]. Briefly, frozen heart tissue was pulverized using a Qiagen TissueLyser II containing pre-chilled metal beads and metabolites were extracted with methanol:chloroform:water (2:1:1 v/v), sonicated, and then centrifuged. Adonitol and ^13^C_3_ lactate were added as internal standards. Metabolite extracts were divided into 2 aliquots for GC–MS analysis and LC–MS analysis. A quality control (QC) sample was generated by pooling supernatants from every sample.

For LC–MS analysis (Agilent 6546 LC/Q-TOF coupled to an Agilent 1290 Infinity II LC), the supernatant was dried and reconstituted in 7:3 v/v acetonitrile and water. Chromatographic separation was performed on an Agilent InfinityLab Poroshell 120 HILIC-Z, 2.1 × 150 mm, 2.7 μm column. The mobile phase consisted of 20 mM ammonium acetate buffer in water (pH = 9.2) (A) and acetonitrile (B). To ensure a constant concentration during gradient elution, the InfinityLab deactivator additive (p/n 5191–4506) was added. Data was acquired in the negative ESI full MS scan mode (scan range: m/z 40 to 1000) using Agilent MassHunter Acquisition software version 10.0. Optimum values for MS parameters were as follows: gas temperature 300 °C; drying gas flow 13 L/min; Nebulizer pressure 40 psi; Sheath gas temperature 350 °C; Sheath gas flow 12 L/min; Capillary voltage 3500 V; Nozzle voltage 0 V; skimmer offset 45 V; Fragmentor 125 V; Octopole 1 RF Voltage 750 V.

Data analysis and peak integration was performed with Agilent MassHunter Quantitative analysis software (version 10.1). An analytical standard mix of glycolytic and TCA cycle intermediates was analyzed along with samples for identification of targeted metabolites in the samples. A custom Agilent Personal Compound Database and Library (PCDL) of target metabolites (glycolytic and tricarboxylic acid cycle intermediates, amino acids and nicotinamide adenine dinucleotide reduced (NADH) and oxidized (NAD^+^) forms) was created from Agilent METLIN PCDL. The retention times derived from analyses of an in-house prepared mix of analytical glycolytic and tricarboxylic acid cycle intermediate standards, a canonical amino acid unlabeled standard mix (Cambridge isotope laboratories, Inc., Cat. No. MSK-CAA-US- 1) were included in the custom PCDL to facilitate the identification of metabolites in the samples. Relative abundance values for target metabolites were obtained by normalizing the raw data with tissue weights and adonitol values.

For GC–MS analysis (Agilent 7890B- 5977 A), the supernatant was dried and derivatized in two steps and the analysis was performed as previously published [8]. Briefly, samples were dissolved in methoxyamine hydrochloride in pyridine for 1.5 h at 37 °C with constant orbital shaking. Bis(trimethylsilyl)trifluoroacetamide (BSTFA) was added and the samples were mixed at 37 °C for 40 min. Derivatized samples were analyzed with the EI source in a scan mode (70–600 m/z). An alkane mix (C10 to C24), an analytical standard mix of glycolytic and TCA cycle intermediates, and a canonical amino acid unlabeled standard mix (Cambridge isotope laboratories, Inc., Cat. No. MSK-CAA-US- 1) were prepared in the same manner as samples. The alkane mix was used as a retention time standard ladder. Data was processed using Mass Hunter Quantitative data analysis software with the integrated mass-spectrometry library from the National Institute of Standards and Technology (NIST, Gaithersburg). The raw data were normalized by tissue weight and adonitol. All raw GC–MS data is available upon request. All chemicals and analytical standards (except for the amino acid standard mix) were purchased from Sigma-Aldrich (St. Louis, MO, USA) except for the pyridine and methoxyamine hydrochloride (Thermo Fisher Scientific, Waltham, MA, USA).

### Proteomics

Heart tissue was flash frozen and kept at − 80 °C. Following pulverization, the powder was resuspended in isolation buffer and centrifuged at 550 × g. 100 µL of the supernatant was mixed with 100 µL 1% SDS and 20 µL 10% SDS, heated, and protein concentrations measured. A volume containing 100 µg of total protein was used for analysis and 100 µL 1% SDS was added, along with 1 µg BSA as an internal standard. The samples were mixed, heated at 70 °C, and precipitated with acetone overnight. The precipitate was reconstituted in Laemmli sample buffer at 1 µg/µL and run 1.5 cm into an SDS-PAGE gel. Each 1.5-cm lane was cut as a complete sample, chopped into smaller pieces, washed, reduced, alkylated, and digested with 1-µg trypsin overnight at room temperature. Peptides were extracted from the gel in 50% acetonitrile. These extracts were dried by Speedvac and reconstituted in 200 µL 1% acetic acid.

For selected reaction monitoring (SRM), a ThermoScientific TSQ Quantiva instrument was used in SRM mode. Previously validated assay panels were used to measure the respective groups of proteins. Most assays were performed monitoring two peptides per protein. A cycle time of 2 s was used to give approximately 15 data points across 30-s chromatographic peaks.

For data independent acquisition (DIA), a ThermoScientific QEx plus instrument was used in the DIA mode with a 20 m/z window working from m/z 350 to 950. The orbitrap was operated at a resolution of 17,500. A full scan spectrum at a resolution of 70,000 was acquired each cycle. These conditions give 7–8 data points across our typical 30-s chromatographic peaks. DIA data were analyzed using the program Skyline based on a large group of internally developed assays, with each using 2–3 peptides, that have been validated in prior experiments.

For both SRM and DIA analyses, Skyline is used to locate and integrate the proper chromatographic peaks. Proper retention times are predicted based on retention time calibration using BSA and trypsin peptides with manual inspection and adjustment as needed. Calculations determine the total protein response from the geomean of the two monitored peptides. Results are normalized to the BSA internal standard and expressed as pmol/100 µg total protein.

### Protein carbonylation assay

Protein Carbonyl content was assessed using a protein carbonyl assay kit according to the manufacturer’s protocol (abcam; ab126287). ~ 35–40 mg of pulverized tissue was used for the analysis.

### Quantitative PCR

RNA was extracted from frozen heart tissue powder (~ 10–15 mg) collected and pulverized in the same manner as samples frozen for metabolomic analysis. The total RNA was extracted using Qiagen RNeasy Fibrous Tissue Mini Kit (Cat. No. 74704) and RNA concentration determined using the Nanodrop 2000 (Thermo Scientific). The reverse transcription reaction was conducted to obtain cDNA using the Quantitect reverse transcription kit (Qiagen) following the manufacturer’s suggested protocol. Quantitative PCRs were performed in duplicates to quantify gene expression levels by using the CFX96 real-time system (Bio-Rad) as directed by the QuantiTect SYBR Green PCR kit (Qiagen). Target gene expression was evaluated using the ΔΔ*CT* method and normalized to the geomean of three reference genes (*Eef1e1*, *Rpl4*, and *Tbp*) for cardiac-specific gene expression analysis in mouse hearts [10, 19]. Previously validated primer pairs were used to quantify p53, p21, p16, IL- 6 mRNA are as follows [20, 21]:

**Table:** 

Gene	Sequence
p53	Forward: 5′-GTATTTCACCCTCAAGATCC- 3′ Reverse: 5′-TGGGCATCCTTTAACTCTA- 3′
p21	Forward: 5′-GTCAGGCTGGTCTGCCTCCG- 3′ Reverse: 5′-CGGTCCCGTGGACAGTGAGCAG- 3′
p16	Forward: 5′-CCCAACGCCCCGAACT- 3′ Reverse: 5′-GCAGAAGAGCTGCTACGTGAA- 3′
IL- 6	Forward: 5′-TGGTACTCCAGAAGACCAGAGG- 3′ Reverse: 5′-AACGATGATGCACTTGCAGA- 3′

### Proteostasis

Aged WT and Glyco^Hi^ mice were administered deuterium oxide (D_2_O), as previously described to determine the synthesis rates of whole homogenate, mitochondrial protein and DNA [5, 22–24]. Briefly, 25–27-month-old mice were intraperitoneally injected with the bolus dose of 99% deuterium oxide (D_2_O) with 0.9% sodium chloride (Sigma-Aldrich) calculated to enrich the body water pool to approximately 5% based on 60% of body weight as water to initiate labelling. Mice were then allowed ad libitum water containing 8% D_2_O for the next 14 days. After a 14-day labelling period, mice were euthanized by isoflurane overdose and blood and heart tissues were harvested rapidly and snap-frozen in liquid nitrogen for later analyses [22]. For measurement of protein synthesis, approximately 30–40 mg of pulverized whole heart tissue was homogenized in isolation buffer (100 mM KCl, 40 mM Tris–HCl, 10 mM Tris Base, 5 mM MgCl2, 1 mM EDTA, 1 mM ATP, pH = 7.6) with phosphatase and protease inhibitors (HALT, Thermo Fisher Scientific) using a bead homogenizer (Next Advance Inc., Averill Park, NY, USA) [5, 23]. The whole tissue homogenate or the mitochondrial fraction, enriched by centrifugation, were used for the subsequent analyses. The pentafluorobenzyl-N,Ndi (pentafluorobenzyl) derivative of alanine was analyzed on an Agilent 7890 A GC (Agilent, Santa Clara, USA) coupled to an Agilent 5975 C MS (Agilent, Santa Clara, USA) as previously described [22, 25].

Body water enrichment was determined as previously described [25]. Briefly,120 μL of plasma was placed in the inner well of an O-ring cap of inverted screw-capped tubes and placed in a heat block for overnight distillation at 80 °C. Distilled samples were diluted 1:300 in ddH_2_O and analyzed on a liquid water isotope analyzer (Los Gatos Research, Los Gatos, CA, USA) against a standard curve prepared with samples containing different concentrations of D_2_O [23, 25].

DNA isolation and DNA synthesis measurement was performed as previously published [22, 24]. DNA was extracted from ∼20 mg pulverized heart tissue (DNA Mini Kit, Qiagen) and the concentration and quality of isolated DNA were determined by NanoDrop (Thermo Fisher Scientific). Next, DNA was hydrolyzed at 37 °C overnight and then acetylated with acetic anhydride and methylimidazole. Dichloromethane extracts were dried, then resuspended with ethyl acetate and analyzed by GC/MS (Agilent 7890 A GC, Agilent 5975 C MS) [22].

To determine the protein and DNA synthesis rates, standard FSR calculations as previously published was used [5, 23, 24, 26]. Briefly, the newly synthesized fraction (f) of proteins was calculated as: Fraction new = *E*_product_/*E*_precursor_, where the *E*_product_ is the enrichment (*E*) of protein-bound alanine and *E*_precursor_ is the calculated maximum alanine enrichment from equilibration of the body water pool. Then the precursor enrichment of alanine was adjusted by mass isotopomer distribution analysis (MIDA) [23].

### Hydroxyproline assay

To determine collagen concentration present in the tissue, we used the hydroxyproline assay as previously published with minor modifications [23]. Briefly, ∼40 mg of tissue and standard hydroxyproline at different concentrations was hydrolyzed by incubation for 24 h at 110 °C in 6 M HCl. Next,10 µL of hydrolyzed sample was mixed with 150 μL isopropanol and 75 μL of chloramine-T (EMD Millipore Sigma, St. Louis, MO, USA) in citrate buffer and oxidized for 10 min at room temperature. The oxidized samples were then mixed with 1 mL of a 3:13 solution of Ehrlich reagent (3 g of *p*-dimethylaminobenzaldehyde (Sigma-Aldrich, St. Louis, MO, USA), 10 mL 100% ethanol, and 675 μL sulfuric acid) to isopropanol and incubated for 30 min at 58 °C [23]. The reaction was stopped on ice for 10 min and quantified by measuring absorbance at 558 nm on a 96-well plate reader in triplicate. Hydroxyproline concentration (μg/mg tissue) was then determined using a standard curve of trans- 4-hydroxy-l-proline (Sigma-Aldrich, St. Louis, MO, USA) [23].

### Statistics

Data are presented as mean ± SD. Statistics were performed with Prism 10 (GraphPad). An unpaired or paired *t*-test was used to compare two groups. When more than two groups were compared, 2-way ANOVA followed by Tukey’s or Sidak multiple comparisons test was performed as indicated. Principal component analysis was performed with PCA plotter V1.02 (https://scienceinside.shinyapps.io/mvda/). Metabolomics and proteomics data were analyzed with MetaboAnalyst 6.0 [27], as described in the figure legends. Statistical significance is indicated in each figure legend.

## Results

### Cardiac function

Our goal was to determine whether constitutively enhanced glycolysis promotes or prevents cardiac pathology and aging. Young Glyco^Hi^ mice have been reported to have cardiac hypertrophy that is accompanied by a mild decrease in ejection fraction [28]. However, in our previous study we did not observe a decline in systolic function between Glyco^Hi^ and WT male 9–11-month-old mice [9]. This suggested that the decline in cardiac function is not progressive.

**Fig. 1:**
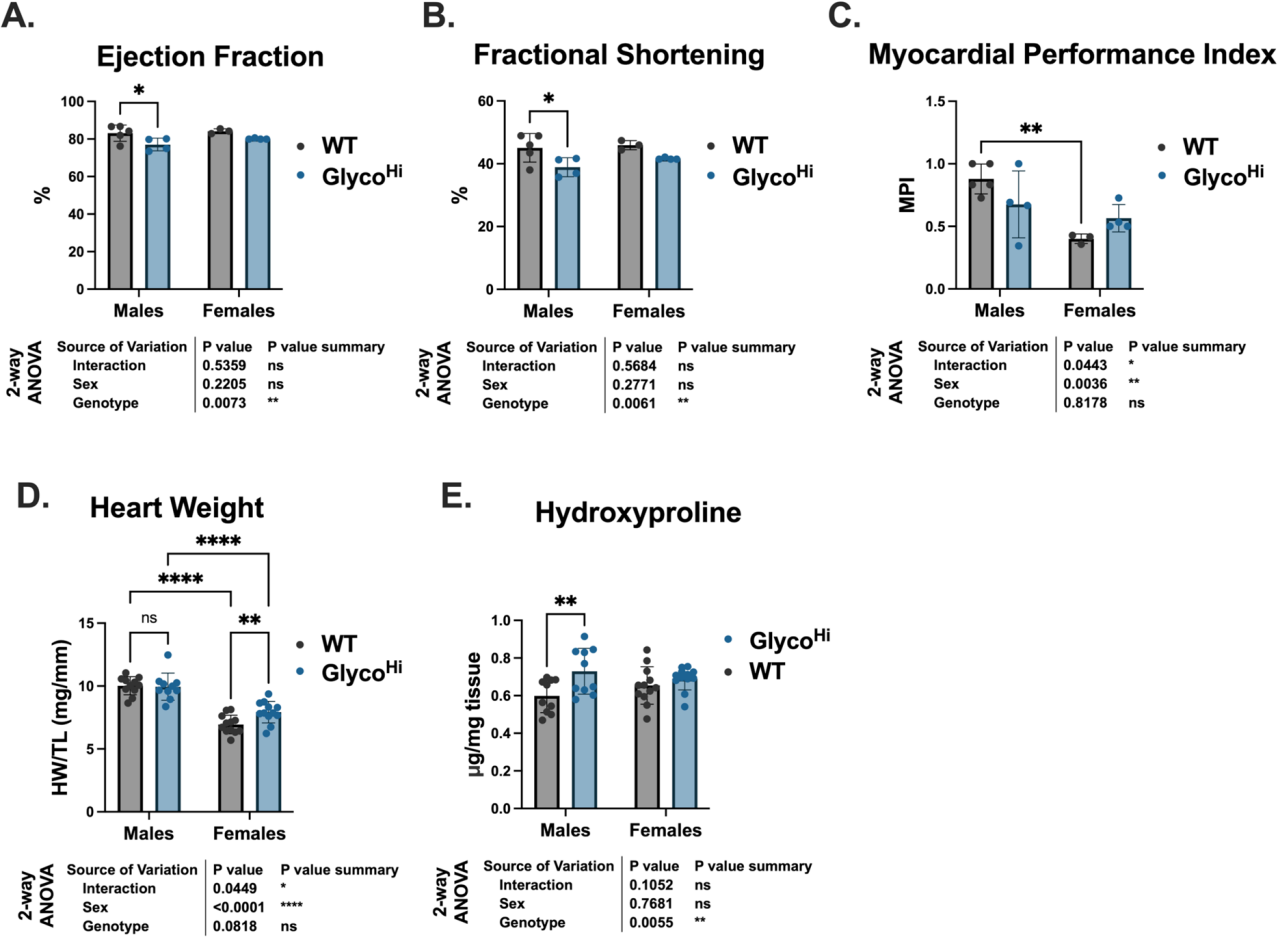
Old Glyco^Hi^ mice have decreased cardiac function regardless of sex, but only females exhibit cardiac hypertrophy. **A** Ejection fraction. **B** Fractional shortening. **C** Female and male myocardial performance index (MPI). **D** Heart weight (heart weight/tibia length). **E** Hydroxyproline content was measured by D_2_O labeling (See Fig. 6). Data are shown as mean ± SD. 2-way ANOVA results show the main effects of genotype, sex, and interaction. Multiple comparisons were performed with Sidak post hoc analysis. *p ≤ 0.05, **p ≤ 0.01, ***p ≤ 0.001; other comparisons were non-significant (ns) (*p* > 0.05)

Here, we measured cardiac function by echocardiography in male and female Glyco^Hi^ and WT mice that were between 21 and 24 months old. Ejection fraction (EF) and fractional shortening (FS), metrics of cardiac contractile function, demonstrated genotypic differences. There were modest but statistically significant decreases in Glyco^Hi^ male, but not female, mice (Fig. 1A, B). The myocardial performance index (MPI), which incorporates both systolic and diastolic functions, showed both sex and interaction effects (Fig. 1C). Multiple comparisons revealed no statistical differences, although the female Glyco^Hi^ mice trended higher than WT (worse performance) while male Glyco^Hi^ MPI’s trended lower (better performance).

As shown in Fig. 1D, there were also sexual dimorphic changes in heart size. There was a significant sex effect with male mouse hearts larger than female mouse hearts. There was also an interaction effect, but no significant genotype effect. Multiple comparisons did reveal female Glyco^Hi^ mouse hearts were significantly larger than WT (7.929 ± 0.86 versus 6.30 ± 1.37 mg/mm), while male mouse hearts were not different between genotypes. This is consistent with our previous report where we showed younger (9–11-month-olds) male WT and Glyco^Hi^ mice had similar heart weights [9]. There were, however, distinct changes in hydroxyproline concentration, a measure of collagen and, a potential contributor to fibrosis (Fig. 1E). Interestingly, male Glyco^Hi^ hearts showed a significant elevation in hydroxyproline concentration relative to WT. In contrast, there was no difference between WT and Glyco^Hi^ female hearts suggesting cardiac hypertrophy is occurring without increased collagen deposition.

**Fig. 2:**
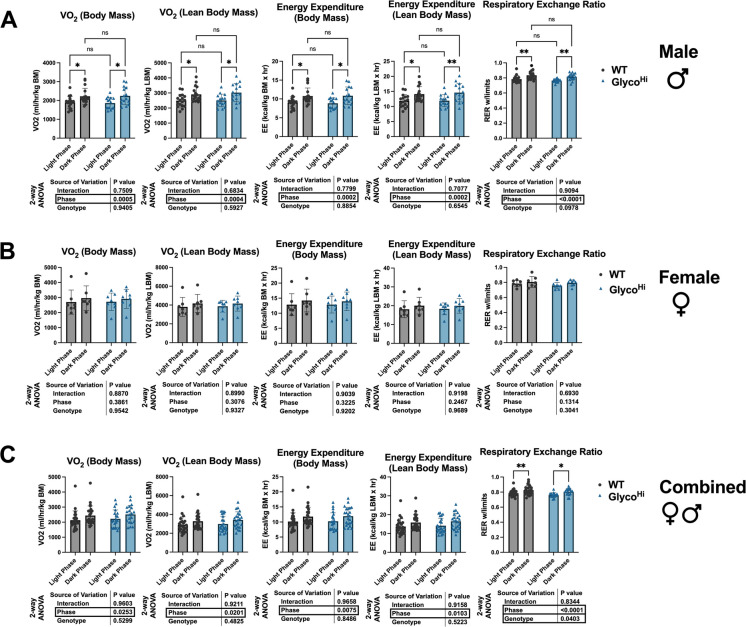
Metabolic cage data of WT and Glyco^Hi^ mice. *Left to Right*: Oxygen consumption rate (OCR) normalized to body weight; OCR normalized to lean body mass; energy expenditure (EE) normalized to body weight; EE normalized to lean body mass; respiratory exchange ratio. **A** Males, **B** Females, **C** Combined sexes. (*n* = 17 WT male; *n* = 7 WT female; *n* = 15 Glyco^Hi^ male; *n* = 8 Glyco^Hi^ female). Data are shown as mean ± SD. 2-way ANOVA results show the main effects of light/dark phase, genotype, and interaction, with significant effects boxed. Multiple comparisons were performed with Sidak post hoc analysis. *p ≤ 0.05, **p ≤ 0.01, ***p ≤ 0.001; other comparisons were non-significant (ns) (*p* > 0.05)

We also performed indirect calorimetry to determine if there were significant differences in overall metabolic substrate utilization and energy expenditure. Body mass and lean body mass were not significantly different based on genotype (Supplementary Fig. 1). Both male WT and Glyco^Hi^ mice showed increased oxygen consumption rates (OCR) and energy expenditure when comparing the dark phase to the light phase (Fig. 2A). This was true whether the data were standardized to total body mass or lean body mass. Likewise, both WT and Glyco^Hi^ mice showed increased respiratory exchange ratios in the dark phase relative to the light phase (Fig. 2A, *far right*). However, no statistically significant differences were seen in any of these parameters based on genotype.

In contrast, female mice regardless of genotype showed no significant increases in oxygen consumption rate, energy expenditure, or respiratory exchange ratio in the dark phase relative to the light phase (Fig. 2B). When data for both sexes was combined, each of the parameters showed a statistically significant effect based on the light/dark phase. RER also showed a difference based on genotype (Fig. 2C, *far right*), suggesting a difference in overall metabolic substrate utilization. Collectively, these data demonstrate similar whole body metabolic parameters between WT and Glyco^Hi^ mice. There is a sexual dimorphism, with females showing less light to dark phase shifts in metabolism, but this occurred in both genotypes.

### Mitochondrial function

**Fig. 3:**
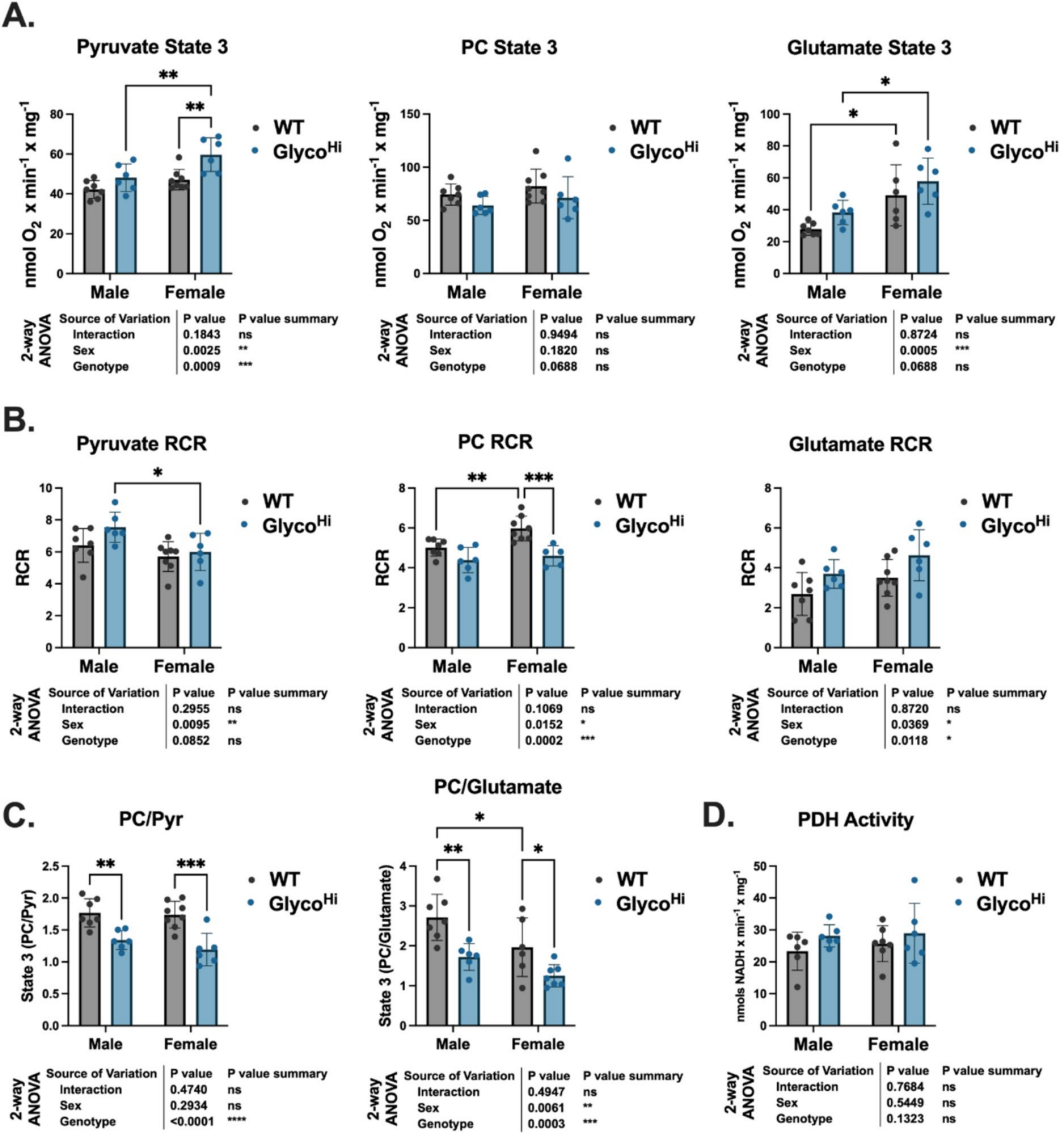
Mitochondrial function and metabolic flexibility differ between aged WT and Glyco^Hi^ mice. **A** The state 3 (ADP-dependent maximal respiration) was measured with isolated mitochondria and the indicated substrates. **B** The respiratory control ratios (RCR, state 3/state 4 rates) are shown for each substrate. **C** The ratio of state 3 respiration rates with the indicated substrates. **D** Pyruvate dehydrogenase (PDH) activity was measured spectrophotometrically, as described in the “Methods”. Data are shown as mean ± SD. 2-way ANOVA results show the main effects of sex, genotype, and interaction. Multiple comparisons were performed with Sidak post hoc analysis. *p ≤ 0.05, **p ≤ 0.01, ***p ≤ 0.001; other comparisons were non-significant (ns) (*p* > 0.05)

Aging is associated with an increase in cardiac glycolysis and a loss of mitochondrial function. The cause-and-effect relationship of these two phenomena are not clear in the aging process. We therefore sought to determine if aged Glyco^Hi^ hearts displayed mitochondrial dysfunction relative to WT. State 3, the maximal rate of ADP-dependent respiration [29], was examined with either pyruvate, palmitoylcarnitine (PC), or glutamate as oxidizable substrates. For pyruvate, 2-way ANOVA revealed both sex and genotype effects with female Glyco^Hi^ showing significantly higher rates as compared to WT females (Fig. 3A, *left*). This supports that Glyco^Hi^ mitochondria are adapted to oxidizing the glycolytic end-product and the results are consistent with our previous reports with young mice [9, 10, 14]. PC supported state 3 respiration was not different based upon genotype or sex (Fig. 3A, *center*). Interestingly, glutamate supported state 3 showed a main effect of sex but not genotype (Fig. 3A, *right*).

The respiratory control ratio (RCR, ratio of State 3 to State 4 respiratory rates) is a classic means of evaluating mitochondrial integrity [30]. A lower ratio indicates less coupling efficiency. With pyruvate as substrate there is a main effect of sex but not genotype (Fig. 3B, *left*). In contrast, for PC there were both genotype and sex effects (Fig. 3B, *center*). Glyco^Hi^ had a lower RCR relative to WT, indicating a lower efficiency for fatty acid supported respiration. Likewise, there were also main effects of sex and genotype with glutamate supported respiration (Fig. 3B, *right*).

To further examine differences in substrate preference, the ratio of PC-supported state 3 to pyruvate-supported state 3 respiration was evaluated [18]. We have previously reported that the ratio of PC- to pyruvate-supported respiration is lower basally in young Glyco^Hi^ mitochondria compared to WT on a normal chow diet [9, 10]. Here, there is a significant genotype effect (Fig. 3C, *left*). For the ratio of PC to glutamate, there were both significant genotype and sex effects (Fig. 3C, *right*). This further supports that Glyco^Hi^ heart mitochondria are metabolically distinct from WT and identifies unique sex differences in cardiac mitochondrial substrate utilization.

Pyruvate dehydrogenase (PDH) activity regulates the overall rate of glucose oxidation. We have previously reported that PDH activity is significantly higher in young Glyco^Hi^ hearts relative to WT [9, 10]. Interestingly, in the aged hearts PDH activity was not significantly different between WT and Glyco^Hi^ groups (Fig. 3D), suggesting a down regulation of PDH activity in Glyco^Hi^ mice with age.

### Metabolic and proteomic phenotypes of aged hearts

**Fig. 4:**
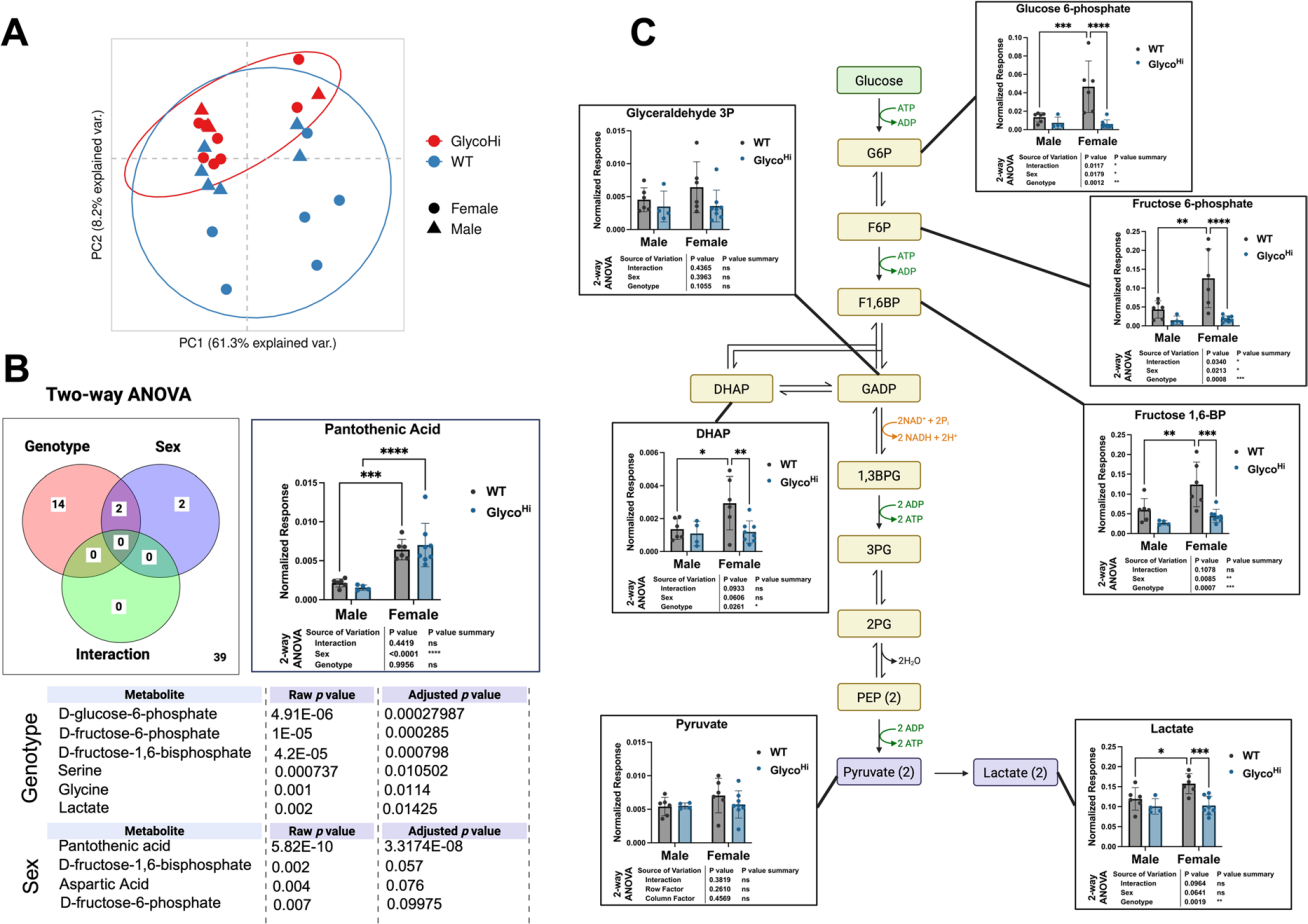
Targeted metabolomics of hearts reveal distinct genotypic differences in glycolytic intermediates. **A** Principal component analysis of metabolomics data from Glyco^Hi^ (*red*) and WT (*blue*). **B** A Venn diagram of 2-way ANOVA results (*left*), based on adjusted *p* values < 0.1 using False Discovery Rate (MetaboAnalyst 6.0). The most significant metabolite that differed by sex is pantothenic acid (*right*). **C** A schematic of glycolysis and the intermediates that were quantified. Multiple comparisons were performed by 2-way ANOVA with Tukey post hoc analysis. Only significant differences are shown: *p ≤ 0.05, **p ≤ 0.01, ***p ≤ 0.001; ****p ≤ 0.0001, other comparisons were non-significant (*p* > 0.05)

We next performed semi-targeted metabolomics to determine how WT and Glyco^Hi^ hearts differed. A combination of LC/Q-TOF and GC/MS identified 57 metabolites. PCA (Fig. 4A, Supplementary Table 1) showed separation between Glyco^Hi^ and WT hearts (*red* and *blue*, respectively) with WT females being the most clearly separated from the other groups (*blue circles*). A Venn diagram of 2-way ANOVA results based on adjusted p-values revealed (Fig. 4B, *left*) shows there were 14 significantly different metabolites based on genotype, 2 based on biological sex, 2 based on sex and genotype, and none that were significantly different based on interactions. Tabular results (Fig. 4B *lower* and Supplementary Table 2) show that 4 of the top 6 most significant metabolites based on genotype are glycolytic intermediates. The two metabolites that have significant differences based on both sex and genotype are fructose- 1,6-bisphosphate and aspartic acid. Pantothenic acid (vitamin B5) was the most significantly different metabolite based on sex. There was a 3.5-fold difference in relative abundance in females versus males regardless of genotype (Fig. 4B, *right*).

Of the 16 significantly different metabolites based on genotype, 5 of them are in the glycolytic pathway (glucose 6-phosphate, fructose 6-phosphate, fructose 1,6-bisphosphate, phosphoenolpyruvate, and lactate) (Fig. 4C). Some metabolites in the pathway (such as pyruvate and glyceraldehyde 3-phosphate) were not significantly different. In agreement with the PCA, the female WT were most distinct, with a higher abundance of several glycolytic intermediates. Reciprocally, Glyco^Hi^ females had a relative decrease in glycolytic intermediates when compared to WT and more closely resembled the levels of metabolites seen in males. This is also in agreement with the PCA where there is more overlap between Glyco^Hi^ males (*blue triangles*) and WT groups. It is thus interesting that while Glyco^Hi^ females have reverted to a more “male metabolic phenotype” they have retained the characteristic elevation in pantothenic acid.

**Fig. 5:**
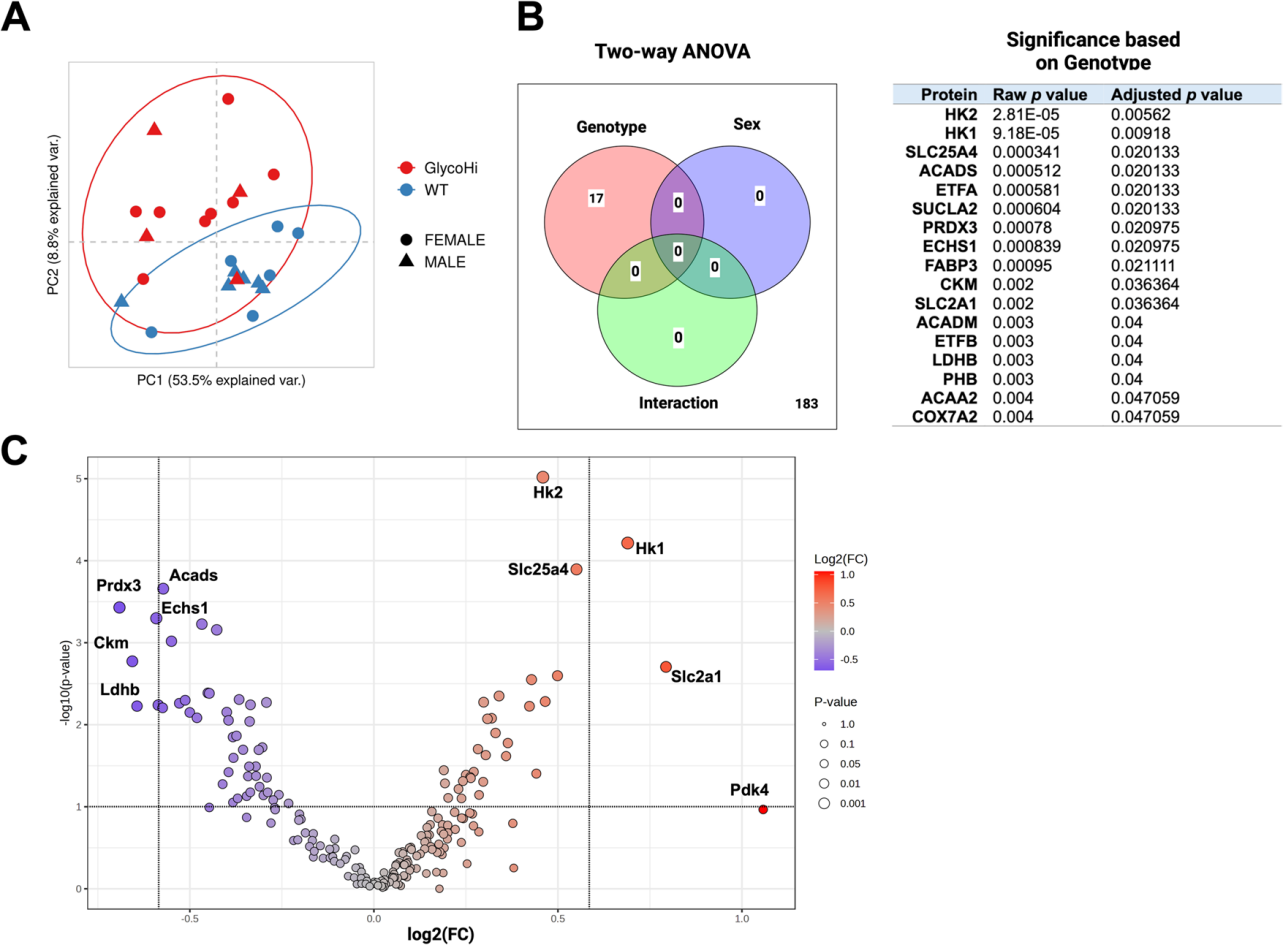
Proteomics of aged hearts reveals genotypic differences. **A** Principal component analysis of metabolomics data from Glyco^Hi^ (*red*) and WT (*blue*). **B** A Venn diagram of 2-way ANOVA results (*left*) that is based on adjusted *p* values < 0.05 using False Discovery Rate (MetaboAnalyst 6.0). The table shows the proteins significantly affected by genotype based on the 2-way ANOVA and adjusted *p*-values. **C** A volcano plot (Glyco^Hi^/WT) was produced in MetaboAnalyst 6.0 using the default settings. Proteins with a positive log2(FC) are relatively more abundant in Glyco^Hi^ than WT. Reciprocally, proteins with a negative log2(FC) are relatively more abundant in WT than Glyco^Hi^

We also performed proteomics on WT and Glyco^Hi^ hearts, quantifying 200 proteins through a selected reaction monitoring methodology (Supplementary Table 3). As shown in Fig. 5A, PCA revealed a clear separation between WT and Glyco^Hi^ proteins. Glyco^Hi^ hearts (*red symbols*) had greater variability than WT. Furthermore, there was no clear separation between male and female groups. Two-way ANOVA analysis revealed all statistically significant differences were due to genotype (Fig. 5B *left*) and are shown in Fig. 5B *right*. A volcano plot comparing Glyco^Hi^/WT hearts (Fig. 5C) showed that the most significant increases were in glycolytic enzymes, including hexokinase 1 (HK1), hexokinase 2 (HK2), and the GLUT1 glucose transporter (SLC2 A1). Reciprocally, WT hearts had more abundant fatty acid oxidation enzymes (ACADS, ECHS1) the antioxidant PRDX3, creatine kinase (CKM) and lactate dehydrogenase (LDHB) when compared to Glyco^Hi^ hearts.

### Hallmarks of aging

**Fig. 6:**
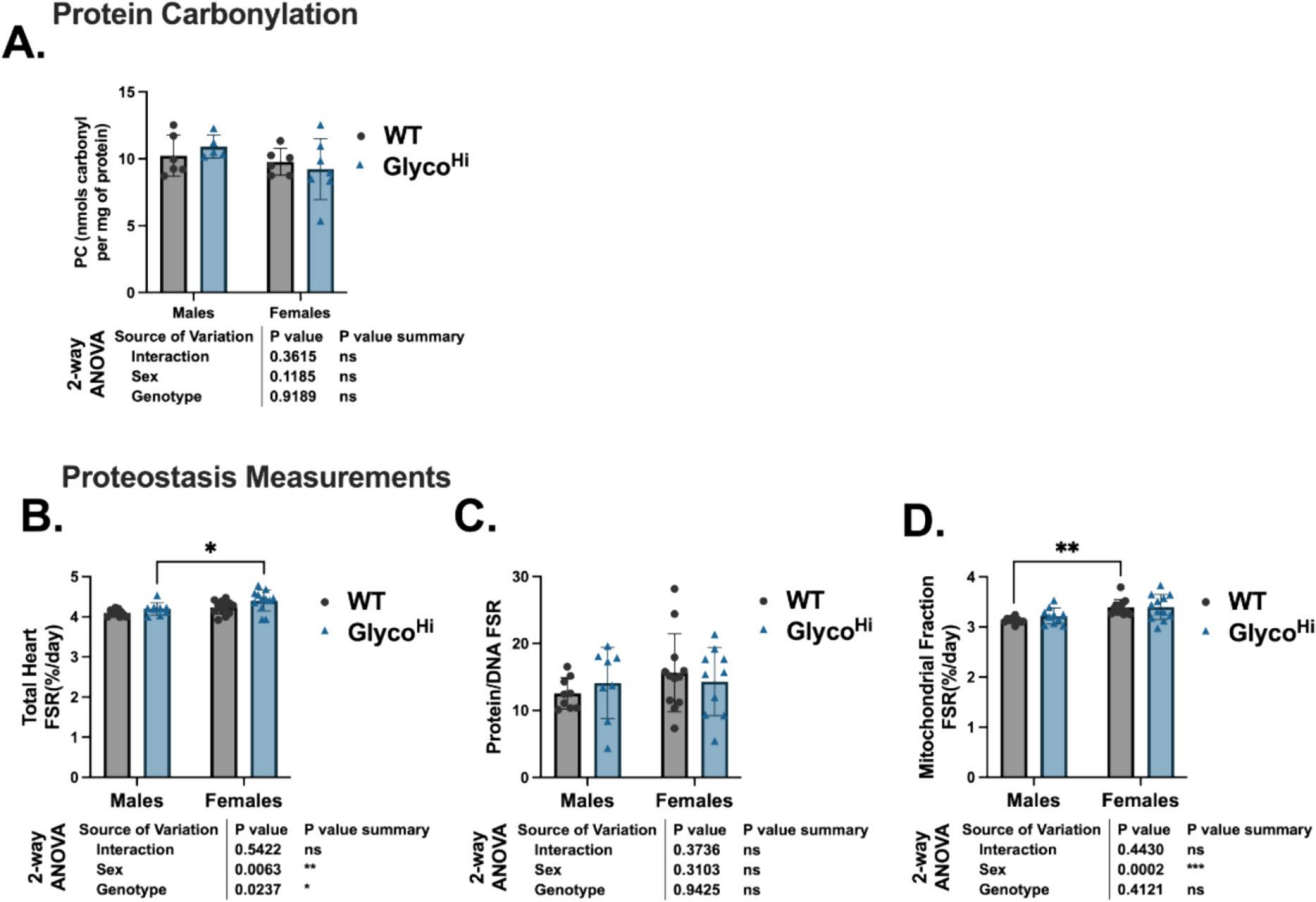
Hallmarks of aging in aged WT and Glyco^Hi^ hearts. **A** Protein carbonylation was assessed in heart tissue homogenates. **B** The fractional synthesis rate (FSR) of proteins in whole heart homogenates. **C** To account for cellular proliferation, the FSR of proteins are presented as a ratio to that of the FSR of DNA. **D** The FSR of proteins in the mitochondrial fraction. Data are shown as mean ± SD. 2-way ANOVA results show the main effects of sex, genotype, and interaction. Multiple comparisons were performed with Sidak post hoc analysis. *p ≤ 0.05, **p ≤ 0.01, ***p ≤ 0.001; other comparisons were non-significant (ns) (*p* > 0.05)

Mitochondrial dysfunction can lead to enhanced ROS production, oxidative damage, and contribute to loss of proteostasis (hallmarks of aging) [31]. Protein carbonyls are a common oxidation product that arise from the oxidation of specific amino acid residues and by the reaction of aldehydes with nucleophilic sites on proteins [32]. Here we measured protein carbonylation in aged male and female WT and Glyco^Hi^ mouse hearts. As shown in Fig. 6A, protein carbonyls were not different between groups. This supports that Glyco^Hi^ mice are not under increased oxidative stress relative to WT.

Cellular senescence is another hallmark of aging [31]. We therefore examined markers of cellular senescence (p16, p21, p53) by quantitative real-time PCR. While p21 showed a statistically significant increase in Glyco^Hi^ hearts relative to WT, p16 and p53 were not different between groups (Supplementary Fig. 2). Furthermore, pro-inflammatory IL- 6, a senescence-associated secretory phenotype (SASP) factor, was expressed at the same levels between groups (Supplementary Fig. 2). These data support that Glyco^Hi^ hearts are not experiencing increased cellular senescence relative to WT.

The biogenesis of new proteins, which is one mechanism of maintaining proteostasis, can be evaluated in vivo by providing animals with deuterium oxide (heavy water, D_2_O) in drinking water and then measuring the incorporation of deuterium into proteins via GC–MS methodology. The fractional synthesis rate (FSR) of new protein is then evaluated on its own or as a ratio to that of the DNA synthesis rate. The latter is done so that the contribution of protein synthesis towards proliferation (growth) versus maintaining existing structures can be evaluated [22, 24, 33]. Aged WT and Glyco^Hi^ mice were administered D_2_O for 14 days, a time point determined from prior experiments that allows for the capture of both short-lived and intermediate-lived proteins. As shown in Fig. 6B, in the total protein homogenate there was a main effect of both genotype and sex on FSR. The data was also analyzed as the ratio of protein FSR to the DNA FSR (Fig. 6C). Evaluating the ratio of protein to DNA FSR’s, the statistically significant differences seen in Fig. 6B were lost. We also measured the FSR of the mitochondrial fraction [34]. Here, there was a main effect of sex, but not genotype, on FSR (Fig. 6D). WT female mice had a greater FSR in the mitochondrial fraction compared to the male mice. Collectively, these data thus show oxidative stress and proteostasis are comparable between aged WT and Glyco^Hi^ hearts.

## Discussion

Metabolic flexibility is essential for normal cardiac function. The heart primarily relies on fatty acid oxidation for its energetic needs but can switch to other nutrients based on demand and nutrient availability. Aging and several cardiac pathologies are associated with a shift from fatty acid oxidation to increased glucose metabolism. However, it is not clear whether an increase in glycolysis is sufficient to drive premature aging. Here we examined old wild type and Glyco^Hi^ mice (21–24 months), which have increased glucose metabolism via the expression of a cardiac-specific transgene (phosphatase-null PFKFB1) that drives the production of fructose- 2,6-bisphosphate and activation of PFK- 1. We demonstrate that increasing cardiac glycolysis results in modest loss of cardiac function, minimal indications of pathology, sustained mitochondrial function, and comparable proteostasis.

Cardiac functional data demonstrate Glyco^Hi^ hearts exhibit modest decreases, relative to WT, in ejection fraction and fractional shortening. In our previous study examining WT and Glyco^Hi^ mice between 9 and 11 months, we saw no difference in these systolic functional parameters [9]. Despite the statistically significant changes in fractional shortening (FS) and ejection fraction (EF), the absolute decreases were relatively modest. EF was 83.5% in WT and 78.6% in Glyco^Hi^; FS was 45.4% in WT and 40.3% in Glyco^Hi^. Overall metabolic substrate utilization and energy expenditure, as shown by indirect calorimetry, were also similar between genotypes. These whole-body metabolism measurements demonstrated similar shifts in metabolic status and energy expenditure in male WT and Glyco^Hi^ between the light and dark phase. One difference is that the effect of genotype is statistically different for RER when analyzed with combined sexes (Fig. 2C, *right*). During the light phase, the average RER of combined sexes for WT was 0.78 ± 0.04 versus 0.75 ± 0.03 for Glyco^Hi^ (*p* = 0.047 by Student’s *t*-test). This raises the possibility that the skeletal muscles and other organs are oxidizing more fat at the expense of increased cardiac glucose oxidation. Unexpectedly, female mice, regardless of genotype, had no significant changes in whole body metabolism when comparing light and dark phases. However, these observations are in line with a previous study that examined different strains of mice and observed weak day–night variations in activity traits, including energy expenditure, in FVB mice regardless of sex [35]. In that study, mice were young (less than 3 months old) as opposed to here (21–24 months). To the best of our knowledge, this is the first study to conduct indirect calorimetry on FVB mice of this age. Others have shown that in the C57BL/6 JOlaHsd mouse strain there is a loss of clear diurnal pattern in aged versus young mice [36]. This is consistent with the known disruption of circadian rhythm that occurs with aging [37].

A lack of cardiac functional differences between aged WT and Glyco^Hi^ could be due to long-term adaptations to sustained glycolysis that normalizes metabolism. However, we also present comprehensive characterization to show Glyco^Hi^ mice do have a unique metabolic phenotype as compared to aged WT mice. This is shown by the targeted metabolomics and proteomics results that demonstrate a separation of groups by PCA that is predominantly driven by genotype. Proteomics results also show differences in several key glucose metabolism enzymes that are relatively more abundant in Glyco^Hi^ relative to WT, including HK1, HK2, and GLUT1. Conversely, WT proteins have relatively higher levels of several enzymes related to fatty acid metabolism. Finally, mitochondrial analyses show Glyco^Hi^ mitochondria have relatively greater ADP-dependent maximal respiration (State 3) with pyruvate as compared to the fatty acid, palmitoylcarnitine. These results are consistent with our previous studies comparing younger Glyco^Hi^ to WT metabolism and mitochondrial functions. One difference, though, is that at younger ages PDH activity was significantly higher in Glyco^Hi^ mice, suggesting over a longer time period compensatory mechanisms may limit the activity of this key regulator of glucose oxidation.

Glyco^Hi^ mice did not exhibit increased hallmarks of cardiac aging as compared to WT. Glyco^Hi^ mitochondria had similar state 3 respiration rates to WT but did differ in substrate preference. Glyco^Hi^ heart mitochondria have higher maximal rates of respiration with pyruvate relative to palmitoylcarnitine but the mitochondrial respiratory control ratio (RCR), a measure of how well mitochondria are coupled, were similar between genotypes (Fig. 3C,F). Furthermore, there were no conclusive differences in cellular senescence markers (p21 was elevated in Glyco^Hi^, but not p16 and p53, Supplementary Fig. 2) or protein carbonylation, a marker of oxidative stress (Fig. 6A). IL- 6 was also unchanged between genotypes, but we did not complete a comprehensive evaluation of SASP given the lack of robust change in cellular senescence markers. We also evaluated protein biogenesis as one mechanism of proteostatic maintenance by using stable isotope labeling in vivo. This methodology has been previously used to evaluate changes in proteostasis with cardiac aging [5, 22]. The occurrence of proteostatic imbalance or proteotoxic stress greatly influences cardiac function due to the high metabolic demand of cardiomyocytes [38]. Here, we show that there is a greater FSR of proteins in the whole heart homogenates (Fig. 6A) that was not evident in the mitochondrial fraction (Fig. 6D). This difference in protein FSR was abolished when normalized to the DNA FSR (Fig. 6E). This finding suggests that the increase in protein FSR is due to cellular proliferation. As cardiomyocytes are terminally differentiated, it suggests proliferation may be derived from other cell types, such as fibroblasts. Indeed, we did observe a modest by statistically significant increase in hydroxyproline concentration (Fig. 6E) in Glyco^Hi^ hearts. Cumulatively, though, these data support proteostatic maintenance is similar between aged WT and Glyco^Hi^ hearts.

Aged male and female hearts are phenotypically different [39, 40]. The inclusion of male and female groups revealed several sex-specific differences here. Functionally, female Glyco^Hi^ hearts trended towards a higher (worse) myocardial performance index, a parameter that incorporates both systolic and diastolic function (Fig. 1C). Furthermore, female Glyco^Hi^ mice exhibited cardiac hypertrophy (Fig. 1D), although this occurred without enhanced hydroxyproline concentration (Fig. 1E), a measurement of collagen. Mitochondrial respirometry revealed sex-dependent differences in substrate oxidation. Both glutamate and pyruvate showed higher state 3 rates in female mice compared to male mice. Furthermore, while PC state 3 rates were unchanged by sex, the RCR (a measure of mitochondrial efficiency) was lower in in female group (Fig. 3B). Metabolomic, but not proteomics, analysis further revealed sex-dependent differences. The most unique group were the aged WT females, as shown by their relative separation from other groups by PCA (Fig. 4A). This is also apparent in examining individual glycolytic intermediates (Fig. 4E), where glucose 6-phosphate, fructose 6-phosphate, fructose- 1,6-bisphophate, and lactate were significantly higher in WT females as compared to the other groups. Interestingly, female Glyco^Hi^ hearts had a metabolomic profile that more closely approximated the male groups (regardless of genotype). The mitochondrial respirometry results and metabolomics are thus consistent with reports showing estrogen can increase cardiac glucose uptake [41], although sex-dependent differences in cardiac metabolism are not completely clear [40]. The metabolite that had the largest sex-based difference, pantothenic acid (vitamin B5), has previously been shown to be more abundant in females relative to males [42]. The functional significance of elevated pantothenic acid, a precursor of Coenzyme A synthesis, in female hearts is not clear but this shows that the sex difference is maintained with age and that its levels are not disrupted by increased glycolytic activity.

Our previous work has shown that PFKFB2 (the cardiac isoform of PFK- 2) is decreased in content the diabetic heart and not properly activated (phosphorylated) in response to adrenergic signals [43]. We showed that PFKFB2 is labile and the enzyme is rapidly degraded in the absence of insulin signaling, and thus becomes chronically decreased in a type 1 diabetes model and with insulin resistance [43, 44]. We have postulated that the loss of PFKFB2 under conditions such as diabetes could lead to decreased metabolic flexibility and contribute to pathology. Indeed, the cardiac specific knockout of PFKFB2 leads to premature death and cardiac dysfunction [14]. Reciprocally, we have shown that the increase in PFK- 2 activity that is in Glyco^Hi^ hearts confers cardioprotection from HFD-induced diastolic dysfunction [9]. The study presented here is thus important because it shows that the continual activation of PFK- 2 activity in Glyco^Hi^ mice does not lead to overt cardiac pathology and premature aging. It is important to note, though, that increasing glucose metabolism through other means (such as increasing the expression of GLUT1 [45] or GLUT4 [46]) does lead to pathology. The excessive uptake of glucose into cardiomyocytes, without increasing glycolytic metabolism, is detrimental [46]. The upregulation of PFK- 2 is unique because it drives the metabolism of glucose through glycolysis, thus minimizing the diversion of glucose into excessive glycogen storage and ancillary glucose metabolism pathways.

In summary, this study investigated the impact of sustained increased glycolysis on cardiac aging and function using Glyco^Hi^ mice. Despite modest declines in cardiac functional parameters such as ejection fraction and fractional shortening, Glyco^Hi^ hearts demonstrated preserved mitochondrial function, minimal pathological changes, and sustained proteostasis. Metabolomic and proteomic analyses revealed distinct metabolic profiles between Glyco^Hi^ and WT hearts, with Glyco^Hi^ hearts exhibiting higher reliance on glucose metabolism and maintained mitochondrial respiratory efficiency. One caveat is that this study did not look at any additive stresses. Thus, it is possible that there is a diminished ability to increase cardiac work (such as induced by high afterload) due to substrate limitations in Glyco^Hi^ mice relative to controls [47]. Another limitation is that we did not exhaustively examine all hallmarks of aging (for example telomere attrition, epigenetic alterations, and chronic inflammation). Nevertheless, our findings do support that increased glycolysis, driven by PFK- 2 activation, does not induce overt cardiac pathology or premature aging, highlighting its potential cardioprotective role compared to other targets of glucose metabolism (such as glucose uptake).

## Supplementary Information

Below is the link to the electronic supplementary material.

Supplementary file1 (DOCX 369 kb)

Supplementary file2 (XLSX 20 kb)

Supplementary file3 (CSV 1 kb)

Supplementary file4 (CSV 29 kb)

## Data Availability

All data supporting the study findings are included in the main and supplementary materials. Raw metabolomics and proteomics data can be found at: 10.5281/zenodo.15232615.

## References

[1] Chiao YA, Rabinovitch PS. The aging heart. Cold Spring Harb Perspect Med. 2015;5(9):a025148. 10.1101/cshperspect.a025148. 26328932 10.1101/cshperspect.a025148 PMC4561390

[2] Chiao YA, Zhang H, Sweetwyne M, Whitson J, Ting YS, Basisty N, Pino LK, Quarles E, Nguyen NH, Campbell MD, Zhang T, Gaffrey MJ, Merrihew G, Wang L, Yue Y, Duan D, Granzier HL, Szeto HH, Qian WJ, Marcinek D, MacCoss MJ, Rabinovitch P. Late-life restoration of mitochondrial function reverses cardiac dysfunction in old mice. Elife. 2020;9. 10.7554/eLife.55513. 10.7554/eLife.55513 PMC7377906 32648542

[3] Lesnefsky EJ, Chen Q, Hoppel CL. Mitochondrial metabolism in aging heart. Circ Res. 2016;118(10):1593–611. 10.1161/CIRCRESAHA.116.307505. 27174952 10.1161/CIRCRESAHA.116.307505 PMC5009371

[4] Mainali N, Ayyadevara S, Ganne A, Shmookler Reis RJ, Mehta JL. Protein homeostasis in the aged and diseased heart. J Cardiovasc Aging. 2023;3(2). 10.20517/jca.2023.4. 10.20517/jca.2023.4 PMC10121184 37092014

[5] Zarzycka W, Kobak KA, King CJ, Peelor FF 3rd, Miller BF, Chiao YA. Hyperactive mTORC1/4EBP1 signaling dysregulates proteostasis and accelerates cardiac aging. Geroscience. 2024. 10.1007/s11357-024-01368-w. 39379739 10.1007/s11357-024-01368-w PMC11979070

[6] Rider MH, Bertrand L, Vertommen D, Michels PA, Rousseau GG, Hue L. 6-phosphofructo-2-kinase/fructose-2,6-bisphosphatase: head-to-head with a bifunctional enzyme that controls glycolysis. Biochem J. 2004;381(Pt 3):561–79. 10.1042/BJ20040752. 15170386 10.1042/BJ20040752 PMC1133864

[7] Wang Q, Donthi RV, Wang J, Lange AJ, Watson LJ, Jones SP, Epstein PN. Cardiac phosphatase-deficient 6-phosphofructo-2-kinase/fructose-2,6-bisphosphatase increases glycolysis, hypertrophy, and myocyte resistance to hypoxia. Am J Physiol Heart Circ Physiol. 2008;294(6):H2889-97. 10.1152/ajpheart.91501.2007. 18456722 10.1152/ajpheart.91501.2007 PMC4239994

[8] Batushansky A, Matsuzaki S, Newhardt MF, West MS, Griffin TM, Humphries KM. GC-MS metabolic profiling reveals fructose-2,6-bisphosphate regulates branched chain amino acid metabolism in the heart during fasting. Metabolomics. 2019;15(2). 10.1007/s11306-019-1478-5. 10.1007/s11306-019-1478-5 PMC6478396 30830475

[9] Mendez Garcia MF, Matsuzaki S, Batushansky A, Newhardt R, Kinter C, Jin Y, Mann SN, Stout MB, Gu H, Chiao YA, Kinter M, Humphries KM. Increased cardiac PFK-2 protects against high-fat diet-induced cardiomyopathy and mediates beneficial systemic metabolic effects. iScience. 2023;26(7):107131. 10.1016/j.isci.2023.107131. 37534142 10.1016/j.isci.2023.107131 PMC10391959

[10] Newhardt MF, Batushansky A, Matsuzaki S, Young ZT, West M, Chin NC, Szweda LI, Kinter M, Humphries KM. Enhancing cardiac glycolysis causes an increase in PDK4 content in response to short-term high-fat diet. J Biol Chem. 2019;294(45):16831–45. 10.1074/jbc.RA119.010371. 31562244 10.1074/jbc.RA119.010371 PMC6851294

[11] Tanner LB, Goglia AG, Wei MH, Sehgal T, Parsons LR, Park JO, White E, Toettcher JE, Rabinowitz JD. Four key steps control glycolytic flux in mammalian cells. Cell Syst. 2018;7(1):49-62 e8. 10.1016/j.cels.2018.06.003. 29960885 10.1016/j.cels.2018.06.003 PMC6062487

[12] Olson AL, Humphries K. Recent advances in understanding glucose transport and glucose disposal. F1000Res. 2020;9. 10.12688/f1000research.22237.1. 10.12688/f1000research.22237.1 PMC7315251 32595948

[13] Dai DF, Karunadharma PP, Chiao YA, Basisty N, Crispin D, Hsieh EJ, Chen T, Gu H, Djukovic D, Raftery D, Beyer RP, MacCoss MJ, Rabinovitch PS. Altered proteome turnover and remodeling by short-term caloric restriction or rapamycin rejuvenate the aging heart. Aging Cell. 2014;13(3):529–39. 10.1111/acel.12203. 24612461 10.1111/acel.12203 PMC4040127

[14] Harold KM, Matsuzaki S, Pranay A, Loveland BL, Batushansky A, Mendez Garcia MF, Eyster C, Stavrakis S, Chiao YA, Kinter M, Humphries KM. Loss of cardiac PFKFB2 drives metabolic, functional, and electrophysiological remodeling in the heart. J Am Heart Assoc. 2024;13(7):e033676. 10.1161/JAHA.123.033676. 38533937 10.1161/JAHA.123.033676 PMC11179765

[15] Griffin TM, Lopes EBP, Cortassa D, Batushansky A, Jeffries MA, Makosa D, Jopkiewicz A, Mehta-D’souza P, Komaravolu RK, Kinter MT. Sexually dimorphic metabolic effects of a high fat diet on knee osteoarthritis in mice. Biol Sex Differ. 2024;15(1):103. 10.1186/s13293-024-00680-6. 39639386 10.1186/s13293-024-00680-6 PMC11619521

[16] Bhaskaran S, Pharaoh G, Ranjit R, Murphy A, Matsuzaki S, Nair BC, Forbes B, Gispert S, Auburger G, Humphries KM, Kinter M, Griffin TM, Deepa SS. Loss of mitochondrial protease ClpP protects mice from diet-induced obesity and insulin resistance. EMBO Rep. 2018;19(3). 10.15252/embr.201745009. 10.15252/embr.201745009 PMC5836096 29420235

[17] Tschop MH, Speakman JR, Arch JR, Auwerx J, Bruning JC, Chan L, Eckel RH, Farese RV Jr, Galgani JE, Hambly C, Herman MA, Horvath TL, Kahn BB, Kozma SC, Maratos-Flier E, Muller TD, Munzberg H, Pfluger PT, Plum L, Reitman ML, Rahmouni K, Shulman GI, Thomas G, Kahn CR, Ravussin E. A guide to analysis of mouse energy metabolism. Nat Methods. 2011;9(1):57–63. 10.1038/nmeth.1806. 22205519 10.1038/nmeth.1806 PMC3654855

[18] Vadvalkar SS, Matsuzaki S, Eyster CA, Giorgione JR, Bockus LB, Kinter CS, Kinter M, Humphries KM. Decreased mitochondrial pyruvate transport activity in the diabetic heart: role of mitochondrial pyruvate carrier 2 (MPC2) acetylation. J Biol Chem. 2017;292(11):4423–33. 10.1074/jbc.M116.753509. 28154187 10.1074/jbc.M116.753509 PMC5377762

[19] Ruiz-Villalba A, Mattiotti A, Gunst QD, Cano-Ballesteros S, van den Hoff MJ, Ruijter JM. Reference genes for gene expression studies in the mouse heart. Sci Rep. 2017;7(1):24. 10.1038/s41598-017-00043-9. 28154421 10.1038/s41598-017-00043-9 PMC5428317

[20] Mohammed S, Thadathil N, Ohene-Marfo P, Tran AL, Van Der Veldt M, Georgescu C, Oh S, Nicklas EH, Wang D, Haritha NH, Luo W, Janknecht R, Miller BF, Wren JD, Freeman WM, Deepa SS. Absence of either Ripk3 or Mlkl reduces incidence of hepatocellular carcinoma independent of liver fibrosis. Mol Cancer Res. 2023;21(9):933–46. 10.1158/1541-7786.MCR-22-0820. 37204757 10.1158/1541-7786.MCR-22-0820 PMC10472095

[21] Thadathil N, Selvarani R, Mohammed S, Nicklas EH, Tran AL, Kamal M, Luo W, Brown JL, Lawrence MM, Borowik AK, Miller BF, Van Remmen H, Richardson A, Deepa SS. Senolytic treatment reduces cell senescence and necroptosis in Sod1 knockout mice that is associated with reduced inflammation and hepatocellular carcinoma. Aging Cell. 2022;21(8):e13676. 10.1111/acel.13676. 35869934 10.1111/acel.13676 PMC9381894

[22] Li P, Newhardt MF, Matsuzaki S, Eyster C, Pranay A, Peelor FF 3rd, Batushansky A, Kinter C, Subramani K, Subrahmanian S, Ahamed J, Yu P, Kinter M, Miller BF, Humphries KM. The loss of cardiac SIRT3 decreases metabolic flexibility and proteostasis in an age-dependent manner. Geroscience. 2023;45(2):983–99. 10.1007/s11357-022-00695-0. 36460774 10.1007/s11357-022-00695-0 PMC9886736

[23] Abbott CB, Lawrence MM, Kobak KA, Lopes EBP, Peelor FF 3rd, Donald EJ, Van Remmen H, Griffin TM, Miller BF. A novel stable isotope approach demonstrates surprising degree of age-related decline in skeletal muscle collagen proteostasis. Function (Oxf). 2021;2(4):zqab028. 10.1093/function/zqab028. 34124684 10.1093/function/zqab028 PMC8187230

[24] Drake JC, Peelor FF 3rd, Biela LM, Watkins MK, Miller RA, Hamilton KL, Miller BF. Assessment of mitochondrial biogenesis and mTORC1 signaling during chronic rapamycin feeding in male and female mice. J Gerontol A Biol Sci Med Sci. 2013;68(12):1493–501. 10.1093/gerona/glt047. 23657975 10.1093/gerona/glt047 PMC3814233

[25] Kobak KA, Lawrence MM, Pharaoh G, Borowik AK, Peelor FF 3rd, Shipman PD, Griffin TM, Van Remmen H, Miller BF. Determining the contributions of protein synthesis and breakdown to muscle atrophy requires non-steady-state equations. J Cachexia Sarcopenia Muscle. 2021;12(6):1764–75. 10.1002/jcsm.12772. 34418329 10.1002/jcsm.12772 PMC8718081

[26] Miller BF, Baehr LM, Musci RV, Reid JJ, Peelor FF 3rd, Hamilton KL, Bodine SC. Muscle-specific changes in protein synthesis with aging and reloading after disuse atrophy. J Cachexia Sarcopenia Muscle. 2019;10(6):1195–209. 10.1002/jcsm.12470. 31313502 10.1002/jcsm.12470 PMC6903438

[27] Pang Z, Lu Y, Zhou G, Hui F, Xu L, Viau C, Spigelman AF, MacDonald PE, Wishart DS, Li S, Xia J. MetaboAnalyst 6.0: towards a unified platform for metabolomics data processing, analysis and interpretation. Nucleic Acids Res. 2024;52(W1):W398–406. 10.1093/nar/gkae253. 38587201 10.1093/nar/gkae253 PMC11223798

[28] Gibb AA, Epstein PN, Uchida S, Zheng Y, McNally LA, Obal D, Katragadda K, Trainor P, Conklin DJ, Brittian KR, Tseng MT, Wang J, Jones SP, Bhatnagar A, Hill BG. Exercise-induced changes in glucose metabolism promote physiological cardiac growth. Circulation. 2017;136(22):2144–57. 10.1161/CIRCULATIONAHA.117.028274. 28860122 10.1161/CIRCULATIONAHA.117.028274 PMC5704654

[29] Chance B, Williams GR. Respiratory enzymes in oxidative phosphorylation. III. The steady state. J Biol Chem. 1955;217(1):409–27. 13271404

[30] Estabrook RW. [7] Mitochondrial respiratory control and the polarographic measurement of ADP: O ratios. Methods in enzymology. Academic Press; 1967. p. 41–7. 10.1016/0076-6879(67)10010-4.

[31] Lopez-Otin C, Blasco MA, Partridge L, Serrano M, Kroemer G. The hallmarks of aging. Cell. 2013;153(6):1194–217. 10.1016/j.cell.2013.05.039. 23746838 10.1016/j.cell.2013.05.039 PMC3836174

[32] Murphy MP, Bayir H, Belousov V, Chang CJ, Davies KJA, Davies MJ, Dick TP, Finkel T, Forman HJ, Janssen-Heininger Y, Gems D, Kagan VE, Kalyanaraman B, Larsson NG, Milne GL, Nystrom T, Poulsen HE, Radi R, Van Remmen H, Schumacker PT, Thornalley PJ, Toyokuni S, Winterbourn CC, Yin H, Halliwell B. Guidelines for measuring reactive oxygen species and oxidative damage in cells and in vivo. Nat Metab. 2022;4(6):651–62. 10.1038/s42255-022-00591-z. 35760871 10.1038/s42255-022-00591-z PMC9711940

[33] Miller BF, Drake JC, Naylor B, Price JC, Hamilton KL. The measurement of protein synthesis for assessing proteostasis in studies of slowed aging. Ageing Res Rev. 2014;18:106–11. 10.1016/j.arr.2014.09.005. 25283966 10.1016/j.arr.2014.09.005 PMC4258117

[34] Hamilton KL, Miller BF. Mitochondrial proteostasis as a shared characteristic of slowed aging: the importance of considering cell proliferation. J Physiol. 2017;595(20):6401–7. 10.1113/JP274335. 28719097 10.1113/JP274335 PMC5638887

[35] Konig C, Plank AC, Kapp A, Timotius IK, von Horsten S, Zimmermann K. Thirty mouse strain survey of voluntary physical activity and energy expenditure: influence of strain, sex and day-night variation. Front Neurosci. 2020;14:531. 10.3389/fnins.2020.00531. 32733181 10.3389/fnins.2020.00531 PMC7358574

[36] Duivenvoorde LP, van Schothorst EM, Swarts HJ, Keijer J. Assessment of metabolic flexibility of old and adult mice using three noninvasive, indirect calorimetry-based treatments. J Gerontol A Biol Sci Med Sci. 2015;70(3):282–93. 10.1093/gerona/glu027. 24615069 10.1093/gerona/glu027

[37] Davidson AJ, Yamazaki S, Arble DM, Menaker M, Block GD. Resetting of central and peripheral circadian oscillators in aged rats. Neurobiol Aging. 2008;29(3):471–7. 10.1016/j.neurobiolaging.2006.10.018. 17129640 10.1016/j.neurobiolaging.2006.10.018 PMC1635489

[38] Guerra J, Matta L, Bartelt A. Cardiac proteostasis in obesity and cardiovascular disease. Herz. 2024;49(2):118–23. 10.1007/s00059-024-05233-6. 38329532 10.1007/s00059-024-05233-6 PMC10917825

[39] Angelini A, Garcia Marquez G, Malovannaya A, Fiorotto ML, Saltzman A, Jain A, Trial JA, Taffet GE, Cieslik KA. Sex differences in response to diet enriched with glutathione precursors in the aging heart. J Gerontol A Biol Sci Med Sci. 2024. 10.1093/gerona/glae258. 10.1093/gerona/glae258 PMC11788829 39492659

[40] Wittnich C, Tan L, Wallen J, Belanger M. Sex differences in myocardial metabolism and cardiac function: an emerging concept. Pflugers Arch. 2013;465(5):719–29. 10.1007/s00424-013-1232-1. 23404619 10.1007/s00424-013-1232-1

[41] Stout MB, Steyn FJ, Jurczak MJ, Camporez JG, Zhu Y, Hawse JR, Jurk D, Palmer AK, Xu M, Pirtskhalava T, Evans GL, de Souza Santos R, Frank AP, White TA, Monroe DG, Singh RJ, Casaclang-Verzosa G, Miller JD, Clegg DJ, LeBrasseur NK, von Zglinicki T, Shulman GI, Tchkonia T, Kirkland JL. 17alpha-Estradiol alleviates age-related metabolic and inflammatory dysfunction in male mice without inducing feminization. J Gerontol A Biol Sci Med Sci. 2017;72(1):3–15. 10.1093/gerona/glv309. 26809497 10.1093/gerona/glv309 PMC5155656

[42] Fulghum K, Collins HE, Jones SP, Hill BG. Influence of biological sex and exercise on murine cardiac metabolism. J Sport Health Sci. 2022;11(4):479–94. 10.1016/j.jshs.2022.06.001. 35688382 10.1016/j.jshs.2022.06.001 PMC9338340

[43] Bockus LB, Humphries KM. cAMP-dependent protein kinase (PKA) signaling is impaired in the diabetic heart. J Biol Chem. 2015;290(49):29250–8. 10.1074/jbc.M115.681767. 26468277 10.1074/jbc.M115.681767 PMC4705931

[44] Bockus LB, Matsuzaki S, Vadvalkar SS, Young ZT, Giorgione JR, Newhardt MF, Kinter M, Humphries KM. Cardiac insulin signaling regulates glycolysis through phosphofructokinase 2 content and activity. J Am Heart Assoc. 2017;6(12). 10.1161/JAHA.117.007159. 10.1161/JAHA.117.007159 PMC5779029 29203581

[45] Yan J, Young ME, Cui L, Lopaschuk GD, Liao R, Tian R. Increased glucose uptake and oxidation in mouse hearts prevent high fatty acid oxidation but cause cardiac dysfunction in diet-induced obesity. Circulation. 2009;119(21):2818–28. 10.1161/CIRCULATIONAHA.108.832915. 19451348 10.1161/CIRCULATIONAHA.108.832915 PMC2765220

[46] Wende AR, Schell JC, Ha CM, Pepin ME, Khalimonchuk O, Schwertz H, Pereira RO, Brahma MK, Tuinei J, Contreras-Ferrat A, Wang L, Andrizzi CA, Olsen CD, Bradley WE, Dell’Italia LJ, Dillmann WH, Litwin SE, Abel ED. Maintaining myocardial glucose utilization in diabetic cardiomyopathy accelerates mitochondrial dysfunction. Diabetes. 2020;69(10):2094–111. 10.2337/db19-1057. 32366681 10.2337/db19-1057 PMC7506832

[47] Ledee D, Portman MA, Kajimoto M, Isern N, Olson AK. Thyroid hormone reverses aging-induced myocardial fatty acid oxidation defects and improves the response to acutely increased afterload. PLoS One. 2013;8(6):e65532. 10.1371/journal.pone.0065532. 23762386 10.1371/journal.pone.0065532 PMC3676337

