# Supplementary material for: The effect of enhanced glycolysis on cardiac aging
Source: GeroScience. 2025 May 1;47(5):6455–72. doi: 10.1007/s11357-025-01656-z (PMC12634992; doi:10.1007/s11357-025-01656-z)

**Supplemental Figure 1.** The body mass and lean body mass were measured by EchoMRI as described in *Methods*. Data are shown as mean ± SD, n=17 WT male, n=15 Glyco^Hi^ male, n=7 WT female, n=8 Glyco^Hi^ female. 2-way ANOVA results show the main effects of sex, genotype, and interaction with significant differences boxed. Multiple comparisons were performed with Sidak post hoc analysis. *p≤0.05, **p≤0.01, ***p≤0.001; ****p≤0.0001; other comparisons were non-significant (ns) (p>0.05).


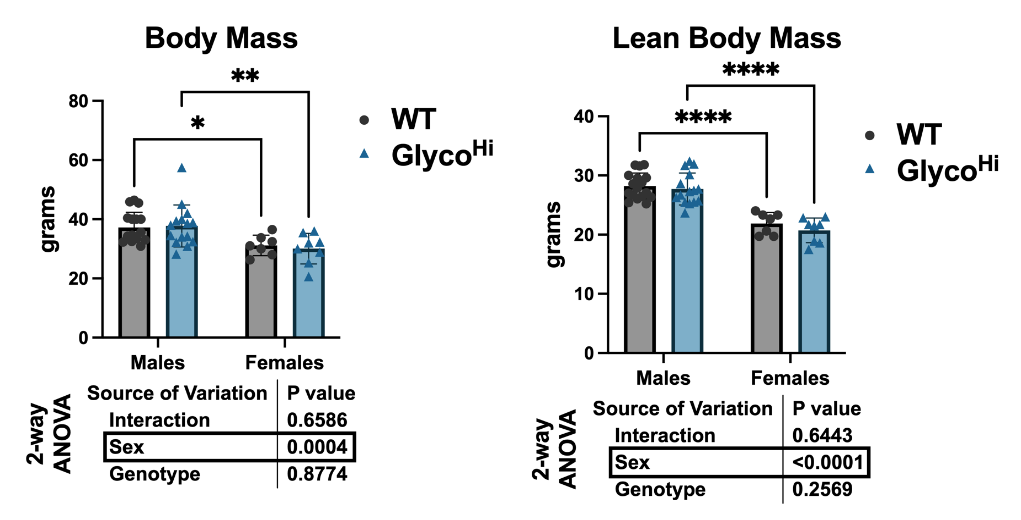


**Supplemental Figure 2.** Cellular senescence markers and IL-6 were measured by quantitative real-time PCR as described in *Methods*. Gene expression is shown as fold change from male WT. Data are shown as mean ± SD, n=5 WT male, n=5 Glyco^Hi^ male, n=5 WT female, n=5 Glyco^Hi^ female. 2-way ANOVA results show the main effects of sex, genotype, and interaction with the significant difference boxed. Multiple comparisons were performed with Sidak post hoc analysis and all comparisons were non-significant (ns) (p>0.05).


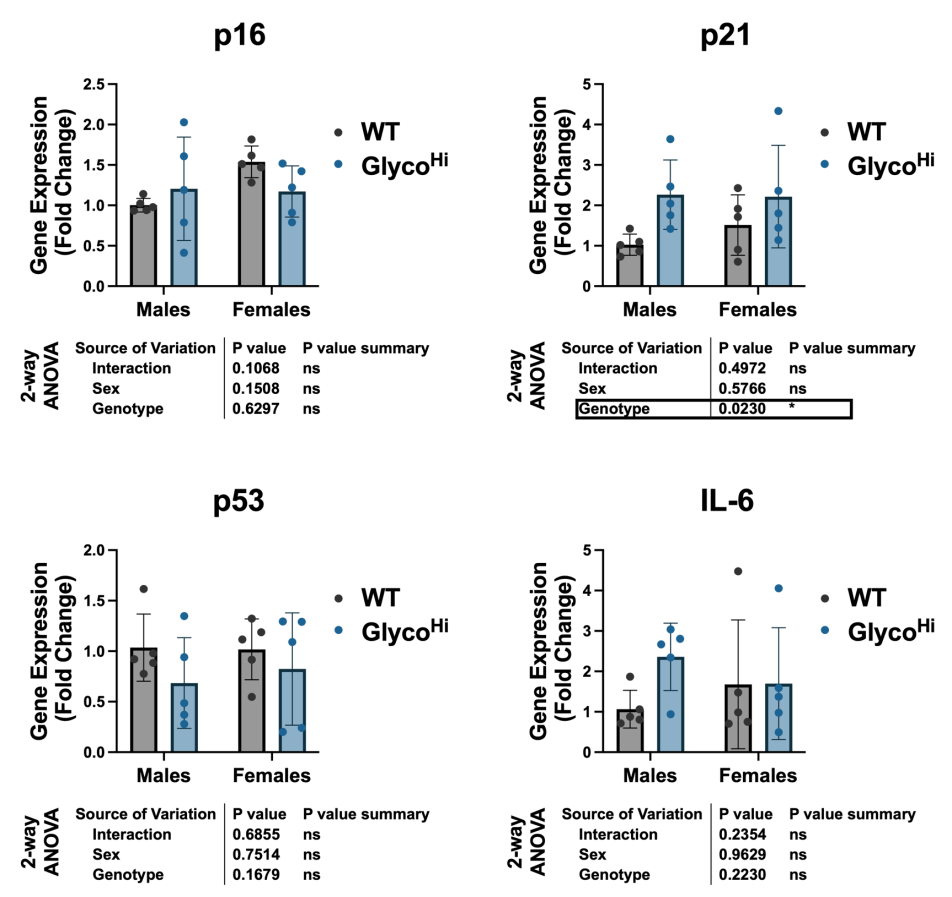

Supplement: Supplementary file 1 — Supplementary file1 (DOCX 369 kb) [file 11357_2025_1656_MOESM1_ESM.docx]
